# Sequence representation as an early step in the evolution of language

**DOI:** 10.1371/journal.pcbi.1011702

**Published:** 2023-12-13

**Authors:** Anna Jon-And, Markus Jonsson, Johan Lind, Stefano Ghirlanda, Magnus Enquist

**Affiliations:** 1 Centre for Cultural Evolution, Stockholm University, Stockholm, Sweden; 2 Department of Romance Studies and Classics, Stockholm University, Stockholm, Sweden; 3 IFM Biology, Linköping University, 581 83 Linköping, Sweden; 4 Department of Psychology, Brooklyn College of CUNY, Brooklyn, New York, United States of America; 5 Department of Psychology, CUNY Graduate Center, New York, New York, United States of America; 6 Department of Zoology, Stockholm University, Stockholm, Sweden; University of Tokyo: Tokyo Daigaku, JAPAN

## Abstract

Human language is unique in its compositional, open-ended, and sequential form, and its evolution is often solely explained by advantages of communication. However, it has proven challenging to identify an evolutionary trajectory from a world without language to a world with language, especially while at the same time explaining why such an advantageous phenomenon has not evolved in other animals. Decoding sequential information is necessary for language, making domain-general sequence representation a tentative basic requirement for the evolution of language and other uniquely human phenomena. Here, using formal evolutionary analyses of the utility of sequence representation we show that sequence representation is exceedingly costly and that current memory systems found in animals may prevent abilities necessary for language to emerge. For sequence representation to evolve, flexibility allowing for ignoring irrelevant information is necessary. Furthermore, an abundance of useful sequential information and extensive learning opportunities are required, two conditions that were likely fulfilled early in human evolution. Our results provide a novel, logically plausible trajectory for the evolution of uniquely human cognition and language, and support the hypothesis that human culture is rooted in sequential representational and processing abilities.

## Author summary

Why only humans have complex language is an unsolved question. Theories of language evolution often highlight the advantage of flexible and precise communication. Given these obvious advantages, it is difficult to explain why language has not evolved in other animals. Here we investigate the hypothesis that the human ability to recognize and remember sequences is an important evolutionary step towards human language, and a key trait for the evolution of human culture and thinking. Mathematical analyses show that remembering and learning to respond to temporal sequences of consecutive events takes a lot of time and is exceedingly costly. This suggests that costs associated with taking sequences into account can explain why language has only evolved once. Computer simulations further show that memory systems found in other animals are more beneficial than sequence memory under most circumstances. Sequence memory is only beneficial when the environment contains information in sequential form, and if individuals are allowed unusually long learning times, conditions fulfilled in human prehistory. Our results suggest a trajectory for the evolution of uniquely human cognition and language, and support the hypothesis that human culture is rooted in memory for stimulus sequences.

## Introduction

Human language is uniquely complex in relation to other species’ communication. Key questions for understanding the evolution of human language are *why* it evolved, why it did *not* evolve in other species, and *what* actually evolved. The question of *why* language evolved is not difficult to answer, considering the enormous advantages of precise and flexible transmission of information for a social species [1–3]. In the light of these advantages, the question of why language has *not* evolved in other species is more difficult to answer, and often left unaddressed. As for the question of *what* actually evolved, theories range from genetically determined linguistic abilities [4–6] to language-specific learning processes [7–9], to claiming that language can emerge from general-purpose learning [10, 11] coupled with cultural processes [12, 13]. Extensive variability across languages in, for example, phonology and grammar, and the gradual learning that requires social input, rule out rigid genetic determination [12, 14]. However, attributing language entirely to learning and culture does not explain why proficient learners like great apes cannot acquire language. The cultural evolution of language must be preceded by the biological evolution of some supporting mental capacities that are not found in other animals [7, 15–17]. Sequential structure is important in language [18–24] and sensitivity to linguistic sequences has been suggested as a fundamental prerequisite for human communication, that may initially evolve as an adaptation to the information structure in foraging environments [25–28].

Here, we explore the simpler hypotheses that domain-general sequence representation is a first step towards human language and thinking, and that non-human animals lack such sequence representation because under most circumstances it is not beneficial. This hypothesis is grounded in a suggested taxonomic gap between humans and other animals in recognizing and remembering sequential information [23, 29, 30]. Our reason for taking this tentative taxonomic gap as our starting point is recent empirical studies showing that animals may not be able to faithfully represent sequential information [23, 30]. Below we expand on this point.

### Sequential abilities in animals

A sequence is here defined as a temporal series of at least two successive stimuli. This can be, for example, a sequence of sounds, sensory input, words in spoken language, or visual observations of events following each other. Faithful sequence representation implies a mental representation with precise information on the order of the stimuli in a sequence. If sequence representation is not faithful, it means that the exact order of the stimuli is not represented, and this information can thus not guide subsequent decisions or behaviour. Recent empirical studies suggest that non-human animals do not rely on faithful sequence representation when discriminating between sequences of stimuli but instead rely on memory traces of stimuli, where the intensity of the memory for each stimulus decays over time. A comprehensive meta-study, incorporating over 100 discrimination experiments in mammals and birds [23] including, for example, rule learning [31, 32], artificial grammar [33–35], sequence discrimination [36, 37] and birdsong [38, 39], shows that the trace memory model can account well for how animals recognize and remember sequences of stimuli, and there are subsequent consistent results from great apes [29, 30]. This points to the importance of considering trace memory as an explanation when limited sequence discrimination is observed in similar studies [40–44]. Importantly, our focus here is on the representation of input stimuli and not on sequential behavioural output. Performing behaviour sequences does not require recognizing and remembering sequential information [45], as it can be learned through primary and conditioned reinforcement [46–48]. Furthermore, computational models that do not rely on sequence representation account well for the acquisition of various behavior sequences in non-human animals, including tool use [49], planning [50], social learning [51] and caching [52].

### Sequences and compositionality in humans and other animals

Compositionality, implying that the meaning of an expression is determined by the meaning of its components and their organization [53], is often considered defining for human language [16, 54]. Linguistic compositionality is open-ended and productive, meaning that humans readily know how and where to insert a new element in a known structure [55]. This is not possible without faithful sequence representation. At the same time, a large body of work in animal cognition and communication claims that a basic form of compositionality can be found in combinations of calls in primates and birds [43, 56–67]. These studies postulate that genetic support underlying relatively simple combinatorial or compositional expressions would be present not only in humans but also in a variety of other species, and many suggest that this provides a key to understanding the evolution of human capacities for more complex and hierarchical compositional structures [68]. There are, however, fundamental differences between combinations of calls in animals and compositionality in human language. Words and morphemes in human languages are learned and arbitrary, allowing for the open-ended productivity that characterizes human language. This kind of open-ended productivity has not been observed in other animals. Processing and producing non-productive call combinations does not require generalized faithful sequence representation. Even vocal learners with the capacity to imitate sound sequences do not recognize and remember arbitrary sequences of information faithfully. Instead, they seem to rely on approximate sequence representation for arbitrary stimuli [23, 30] and specialized memory mechanisms for vocal learning [69, 70]. Thus, while there are surface similarities between combinatorial communication in animals and humans, it is not clear that they rely upon similar biological foundations. This motivates our theoretical investigation of the alternative hypothesis that faithful sequence representation is a domain-general prerequisite for the human language ability that is not found in other animals. This hypothesis aligns with the view that language structure is culturally emergent rather that inborn, a view prevalent in cognitive linguistics and with broad support in the field of language evolution [13, 71–78]

### A hypothesis for language, culture and thinking

Considering the general nature of the tentative taxonomic gap related to sequence representation, prerequisites for language may also underlie other phenomena. Many fundamental human capacities require the ability to represent, store and recall sequential information and develop gradually from an early age, such as sequence imitation [79, 80], causal understanding [81], planning [82, 83], mathematics [84, 85], music syntax [86], and reading and writing [87]. Human sequence processing capacities may thus provide a starting point for understanding the evolution of uniquely human cognitive elements including not only language but also thinking and cumulative culture on a grand scale [88]. Sequence representation as a necessary evolutionary step towards language constitutes an explicit hypothesis aiming at answering the question of *what* evolved. This hypothesis also has the potential to explain why language has *not* evolved more than once, given that generalized sequence representation, as we will show, is not only beneficial but also very costly.

### Benefits and costs of sequential information

Before considering the evolution of memory capacities we want to emphasize that they incur costs. Consider an organism that can perceive *n* different stimuli in the world. As we are investigating the costs of a general sequence memory we are not constrained to linguistic or communicative stimuli, but refer to any kind of stimulus that can be seen, heard, felt, smelled or tasted by the organism in its environment. If the organism makes decisions based only on the last perceived stimulus, it only needs to learn to recognize and respond to *n* situations. If, however, the organism considers the two last perceived stimuli, it has to learn to respond to up to *n*^2^ situations, which requires more time and effort. In general, representing the last *ℓ* stimuli means having to learn to respond to up to *n*^*ℓ*^ situations, which means that increasing *ℓ* generates exponentially increasing learning costs. In reality, not all of these sequences are likely to occur, but even if only a fraction of them do, increasing *ℓ* will still generate accelerating growth of the number of sequences. If the number *n* of perceived single stimuli is constant, these costs are determined purely by the sequence length considered for decision making, even if a shorter length suffices for productive behavior. For example, suppose that the current stimulus is sufficient to behave productively, and for simplicity we consider all possible combinations of stimuli. An organism that can take into account the current stimulus and the previous one will still have to decide what to do in *n*^2^ situations, even if it eventually will learn the same behavior in all sequences that end with the same stimulus. This is because two-stimulus sequences such as (*A*, *B*) and (*C*, *B*) will appear different, and the fact that they require the same behavior (determined by the *B* stimulus) will need to be discovered by trial and error. Representing longer sequences is also likely to incur increased costs related to memory and processing time, but we do not consider these costs in our analysis in order to keep the model simple and to focus on learning costs. In this manuscript we study the benefits and costs of representing input sequences faithfully. We first explore the general costs of sequential information and its relation to learning opportunities and information distribution in an analytical model. We then proceed to investigate the performance of different strategies for representing sequences in learning simulations, where learners are exposed to environments with different information distributions and information structures that we consider more typical for non-cultural and cultural information respectively.

## Results and discussion

### Learning costs may prevent sequence representation from evolving

To explore a potential first step in the evolution of language we use both analytical modelling and computer simulations of learning. For a detailed description of the computer simulations and the relation between the simulations and the analytical model, see the Methods section.

To understand when evolution would favor taking sequential information into account, we start by investigating the utility of sequential information in an analytical model. The purpose of the model is to gain a general understanding of the learning costs associated with the combinatorial explosion that comes with sequential information. As stated above, this combinatorial explosion is generated by the fact that if an organism can perceive *n* stimuli in the world and the same organism can consider the *ℓ* last perceived stimuli when making a decision, the organism will perceive up to *n*^*l*^ different situations and has to learn the best response to each of them. The question is, given this assumption, what circumstances would be necessary for representation of sequences to be beneficial? We address this question in a formal analysis.

To understand when evolution would favor taking sequential information into account, we estimate as follows the fitness of an organism that uses the last *ℓ* stimuli to make decisions. We call *ℓ* the decision depth, that we assume to be constant within each individual. We label a decision “productive” if it is the option that yields the highest utility, e.g. eating when seeing food or answering “yes” when asked if you want dinner. Making a non-productive decision implies losing time and energy. Fitness is defined as the expected number of productive decisions over a lifetime, say *T* time steps. Time is stepped at each sequence exposure. This means that at time *t* the organism has been exposed to *t* sequences. If *u*(*ℓ*, *t*) is the probability that the decision taken at time *t* is productive, given a decision depth of *ℓ*, then fitness is:

$$\begin{array}{c} U\left(\ell ,T\right)=\sum_{t=0}^{T-1}u\left(\ell ,t\right) \end{array}$$ (1)

We calculate *u*(*ℓ*, *t*) based on two factors: whether a productive decision is possible, in principle, based on the last *ℓ* stimuli, and whether the organism actually has learned to make this decision. To formalize the first factor, we denote by *f*(*ℓ*) the fraction of sequences of length *ℓ* in the environment that contains sufficient information for a productive decision. Note that a sequence that contributes to *f*(*ℓ*) also contributes to *f*(*ℓ* + 1): if a productive decision is possible using the last *ℓ* stimuli, then it is also possible using the last *ℓ* + 1 stimuli. In summary, *f*(*ℓ*) increases monotonically with *ℓ* and describes how increasing decision depth increases the organism’s potential to make productive decisions. The extent of this increase is determined by the temporal distribution of information (see examples below).

To formalize how organisms learn productive decisions, we first assume no innate knowledge, such that *u*(*ℓ*, 0) = 0. Let *τ* be the number of experiences needed to learn a single productive decision, and let *N*(*ℓ*) be the number of sequences of length *ℓ* that can be encountered. We assume that *u*(*ℓ*, *t*) increases at each time step according to:

$$\begin{array}{c} u\left(\ell ,t+1\right)=u\left(\ell ,t\right)+\frac{1}{\tau N(\ell )}[f(\ell )-u(\ell ,t)] \end{array}$$ (2)

The motivation for Eq 2 is as follows. The maximum that *u*(*ℓ*, *t*) can increase at any time *t* is 1/*N*(*ℓ*), because at time *t* the animal can learn a productive response to at most one out of *N*(*ℓ*) sequences, and becaue *u*(*ℓ*, *t*) is the fraction of sequences with a known productive response. This maximum increase, however, is typically not realized. First, learning a response requires *τ* experiences, such that the average increase in one experience is only 1/*τ* of the maximum. Second, *u*(*ℓ*, *t*) can increase only if a productive response is not already known to the sequence experienced at time *t*, and the probability of this happening is *f*(*ℓ*) − *u*(*ℓ*, *t*).

The nonhomogeneous first-order linear recurrence (in *t*) in (2) is solved through standard techniques using the initial condition *u*(*ℓ*, 0) = 0. The solution is $u\left(\ell ,t\right)=f\left(\ell \right)[1-{\left(1-\frac{1}{\tau N(\ell )}\right)}^{t}]$. Inserted into (1) this yields

$$\begin{array}{c} U(\ell ,T)=f\left(\ell \right)[T-\tau N\left(\ell \right)(1-{\left(1-\frac{1}{\tau N(\ell )}\right)}^{T})]. \end{array}$$ (3)

**Fig 1:**
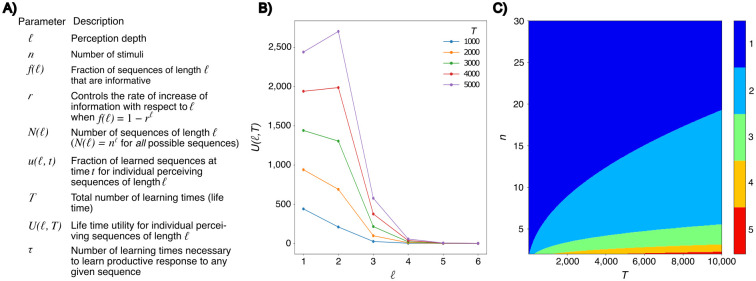
Costs and benefits of considering sequential information in learning and decision making. Costs and benefits of considering sequential information in learning and decision making. **a**: Parameter description for the model. **b**: The utility function *U*(*ℓ*, *T*) visualized for sample values of *T* with *n* set to 12. **c**: Optimal decision depth *ℓ* when *T* and *n* vary. In both (a) and (b) *r* is set to 0.5 *τ* is set to 10. For visualization of the effect of variation in *r* and *τ*, see S1 File.

To study the optimal decision depth *ℓ*, we need concrete assumptions for *N*(*ℓ*) and *f*(*ℓ*). We assume that sequences are formed by selecting randomly from a set of *n* stimuli (with replacement), yielding *N*(*ℓ*) = *n*^*ℓ*^ (Fig 1A). We also assume that *f*(*ℓ*) (the fraction of sequences of length *ℓ* that admits a productive response) changes with *ℓ* in the following way:

$$\begin{array}{c} f\left(\ell \right)=1-r^{\ell } \end{array}$$ (4)

where 0 < *r* < 1. This function increases with *ℓ*, meaning that increasing decision depth increases the potential for productive decisions. However, when *r* is large (close to 1) the increase is slow, enabling us to model environments that favor either small or large decision depth.

Fig 1B and 1C shows that, under a majority of conditions, the maximum of *U*(*ℓ*, *T*) is achieved for *ℓ* = 1. The main reason is that the number of possible sequences, *n*^*ℓ*^, is very large even for modest values of *n* and *ℓ*. This means that the cost of increasing *ℓ* is prohibitive even when the number of learning experiences is large. For example, with *T* = 10, 000 learning experiences, *ℓ* = 2 is favored over *ℓ* = 1 only when *n* < 20 (Fig 1C), which is exceedingly small compared to the number of stimuli realistically encountered by animals.

Since not all of the *N*(*ℓ*) = *n*^*ℓ*^ theoretically possible sequences can be realized, one may scale this number by some constant factor *α*. However, as we see in Eq (3), *N*(*ℓ*) always occurs scaled with *τ*, so we may integrate the *α*-scaling of *N*(*ℓ*) into the existing *τ*-scaling. In the S1 File, an analysis of the effect of varying *τ* to this analytical model can be found.

To further illustrate the combinatorial explosion and resulting learning costs, we have also simulated learning scenarios where learners have varying decision depths. In the learning simulations, similarly to the analytical model, the decision depth *ℓ* determines the length of the sequence of recently perceived stimuli that are considered when making a decision (see the Methods section for details). We call the learners representation of sequences a *Depth-ℓ* representation [89].

**Fig 2:**
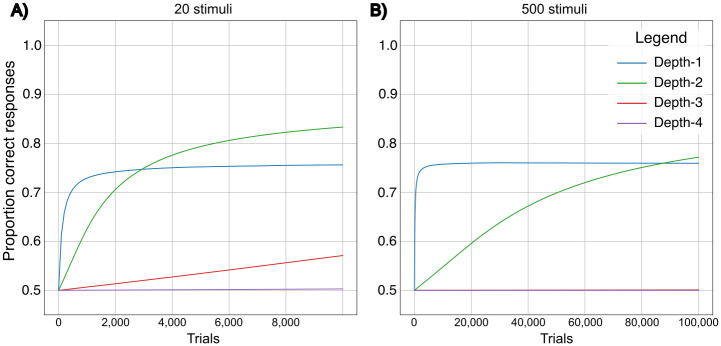
Performance of *Depth-ℓ* representations of stimulus sequences in environments of different sizes. The x-axis represents the time-steps or learning opportunities and the y-axis represents the performance measured after a given number of time-steps, as described in the methods section. **a**: Learning in an environment consisting of 20 different stimuli. **b**: Learning in an environment consisting of 500 different stimuli. In both environments, the rate of increase of information with respect to the increase of *ℓ* is 0.5 (approximating the parameter setting *r* = 0.5 in the analytical model). However, the information increase ceases when *ℓ* > 4, as we are only including *Depth-*1 − 4 representations in the simulations. The learning rate in the simulations approximates *τ* = 10 in the analytical model.

Simulations show that learning is initially much faster with smaller decision depths (Fig 2), and results correspond qualitatively well to those of the analytical model. This is due to the fact that, just like in the analytical model, the number of sequences that the individual needs to learn to respond to grows exponentially when decision depth *ℓ* increases.

In the simulated examples we have used conservatively small worlds, containing between 0 and 30 stimuli (Figs 1 and 2A), while most animals need to learn about many more stimuli. If we increase the number of stimuli to 500, still a conservative number, we see that after around 5, 000 trials, a *Depth-*1 representation supports optimal responses to approximately 75% of the sequences it encounters, while it takes a *Depth-*2 representation over 80, 000 trials, i.e. 16 times as long, to reach the same performance. The analytical model and simulations both point to the learning costs of decision depths of *ℓ* > 1, that may potentially prevent sequence representation from evolving. They also show that remarkably long learning times are required to overcome these costs.

### Approximate sequence representations can decrease learning costs

The result that learning about stimulus sequences is too costly to be practical is counterintuitive, because many animals are sensitive to stimulus sequences to some extent, and because stimulus sequences can be very informative in natural environments. For example, a bird can continue to pursue a bug that has disappeared under a rock, even if now it can only see the rock. We suggest that animals, in general, represent sequences approximately as a compromise between avoiding learning costs and retaining information. The combinatorial cost of learning stimulus sequences can be reduced by ignoring the order in which stimuli occur, and simply consider the identity of the last few stimuli [25]. A strategy that reduces combinatorial costs in a similar way and at the same time contains some sequential information is a “trace memory” representation. This representation has no definite length, rather, stimuli farther back in the past are remembered more faintly. There is no explicit indication of when a stimulus has occurred, but because of the exponential fade of the memory traces, there is a positive correlation between the strength of the memory trace and the recency of the perception of the stimulus. The trace memory is well documented, and it is surprisingly powerful, including a limited ability to support discrimination between stimulus sequences that fits with animal data [23, 90–92]. This is because it focuses on current stimuli and at the same time allows information about the immediate past to be recruited when needed. In the following learning simulations we compare the efficiency of a trace memory representation (see the Methods section for details) to the previous *Depth-ℓ* representations.

**Fig 3:**
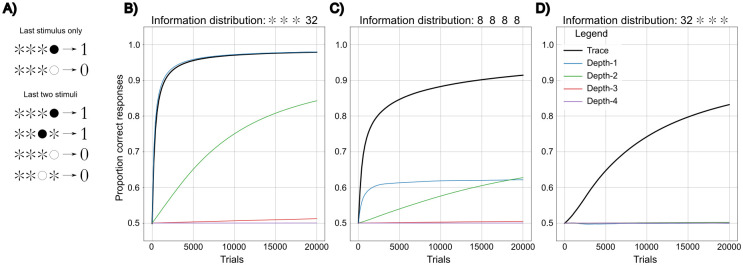
Performance of *Depth-ℓ* and trace representations of stimulus sequences in environments that vary in the temporal distribution of information. The number of stimuli (including informative and uninformative stimuli) is 66 in all environments. The trace decay rate *θ* = 0.5. The x-axis represents the time-steps or learning opportunities and the y-axis represents the performance measured after a given number of time-steps, as described in the methods section. **a**: Examples of environments in which productive decisions depend on the last stimulus only (top) or on the last two stimuli (bottom). ✲ indicates uninformative stimuli selected at random for each pattern; ● and ◯ indicate stimuli whose identity determines the correct output. 1 and 0 indicate whether a response is productive or not. **b**: Learning in an environment of 32 sequences in which only the last stimulus is informative. **c**: Learning in an environment of 32 sequences in which all four temporal positions are equally likely to be informative. **d**: Learning in an environment of 32 sequences in which only the first of the four temporal positions is informative.

We simulate learning in three environments that differ in the temporal distribution of information (Fig 3A). If all information is in the last stimulus, the *Depth-*1 representation, that only considers the last stimulus, is naturally the most efficient learner, but the difference between *Depth-*1 and a trace memory is very small (Fig 3B). This is because the last stimulus is represented with greater intensity than the other stimuli by the trace memory, making it easy for the trace memory to learn to ignore the previous noise stimuli. As soon as some information is in the past, the approximate sequence representation of the trace memory is more efficient than the accurate *Depth-ℓ* sequence representations. *Depth-ℓ* representations generate very high learning costs as *ℓ* increases, in correspondence with our previous cost-benefit analysis. An even information distribution over four time steps clearly favours trace memory (Fig 3C), and even when all information is four steps back in time, a trace memory is much more efficient than a *Depth-*4 representation Fig 3D). The efficiency of trace memory may explain why most animals appear to adopt similar memory strategies [70]. In conclusion, a trace memory is a powerful and productive compromise between information accuracy and learning efficiency that may serve most needs in nature, and that may potentially prevent more accurate sequence representations from evolving.

### Evolution of accurate sequence representations

Despite its efficiency, a trace memory has several limitations that makes it insufficient for human language and other mental abilities that require accurate sequence representations. A trace memory is not useful to learn about longer sequences and it has difficulties with information that is tied to the relative position of stimuli. For example, discriminating between (*A*, *B*) vs. (*B*, *A*), is important for comprehending the meaning of linguistic expressions at all levels, from phonetics to discourse (see Table 1). The sequences (*A*, *B*) and (*B*, *A*), however, can generate similar traces depending on stimulus duration, thereby preventing learning to tell the two sequences apart. For example, a long *A* followed by a short *B* can result in a similar representation to a short *B* followed by a long *A*, so that recovering the order of *A* and *B* may be impossible [23]. Although structure is often more important than order in language [4, 6, 93], representing order is necessary for establishing the structure of many linguistic expressions. How could a machinery evolve, that represents input sequences with enough precision to support language? Two requirements have to be fulfilled. First, such a machinery must develop a sensitivity towards the relative position of stimuli. Second, learning costs must be kept lower than those of *Depth-ℓ* representations, for the combinatorial reasons shown in the above analyses.

**Table 1:** Sequential order and meaning in language.

Linguistic unit	*A*, *B*	*B*, *A*
phoneme	[it] (eat)	[ti] (tea)
syllable	[Ə.raƱnd] (around)	[raƱnd.Ə] (rounder)
word	killer whale	whale killer
sentence	I entered the house. It was cold.	It was cold. I entered the house.

Examples from English of linguistic expressions or parts of discourse with two elements whose order determines meaning, at the phoneme, syllable, word and sentence level. It is mainly words, or morphemes, that constitute the basis for linguistic compositionality, but sequential order is important at both lower and higher linguistic levels.

**Fig 4:**
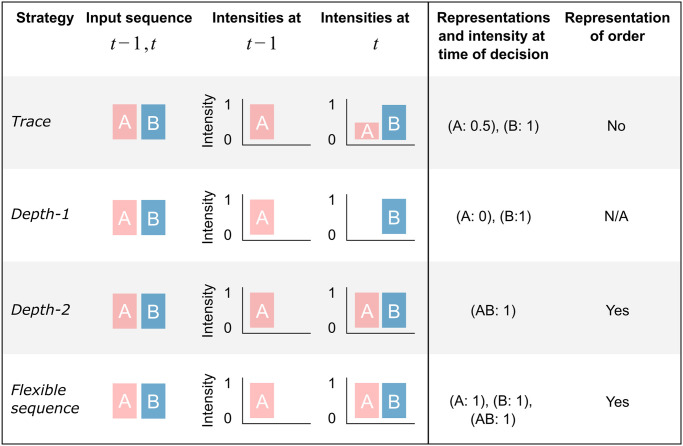
Summary of representation strategies. This illustrates how an input sequence (*A*, *B*) is represented differently by four strategies, and thus generates different representations on which each respective decision on response is based. The *Trace* strategy represents *B* and also a trace of *A* that has faded in intensity from 1 to 0.5 according to the decay rate *θ* = 0.5. The *Depth-1* strategy only represents *B* at the time of decision. The *Depth-2* and *Flexible Sequence* strategies represent *A* and *B* with full strengths and their order, at the time of decision. The *Depth-2*establishes a unique representation of the full sequence (*A*, *B*). The *Flexible Sequence* strategy establishes the same representation of the sequence (*A*, *B*) but also represents sub-sequences, here the single stimuli, thus enabling decision making based on any of these representations.

In order to test if the extreme learning costs that come with *Depth-ℓ* representations can be reduced by an accurate but more flexible sequence representation, we complement *Depth-ℓ* with the ability to represent all substrings of length < *ℓ*. A *Flexible sequence* representation of the stimulus sequence (*A*, *B*, *C*) includes the representation of the individual stimuli *A*, *B*, and *C* and the combinations (*A*, *B*), (*B*, *C*), and (*A*, *B*, *C*) (for more details, see the Methods section). The *Flexible sequence* representation echoes suggestions that humans can encode “chunks” of information of different lengths within the limits of working memory [25, 94–98]. Furthermore, if sequence representation and flexible chunking are used recursively, they allow for processing of hierachical linguistic structure [99]. For a summary of all the different simulated representation strategies, see Fig 4.

**Fig 5:**
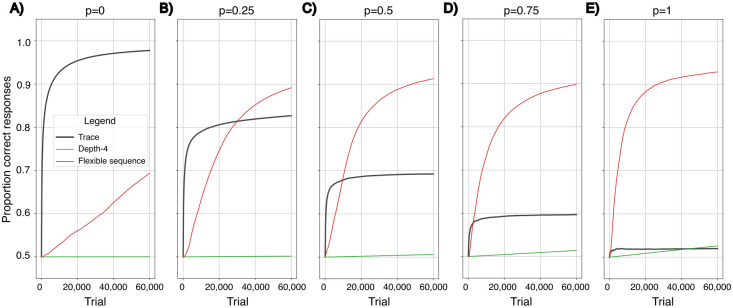
Performance of *Flexible Sequence, Depth*-4 and *Trace* representations, in environments with varying proportions of sequentially structured information. For the *Flexible Sequence* and *Depth-*4 representations *ℓ* = 4. For the trace representation *θ* = 1/2. The probability of encountering information in sequences is determined by *p* in each environment. Sequential information is contained in the two sequences (*A*, *B*) and (*B*, *A*) that are equally distributed over the three time steps where they fit. All other information is in single stimuli and is equally distributed over the four time steps. The x-axis represents the time-steps or learning opportunities and the y-axis represents the performance measured after a given number of time-steps, as described in the methods section. **a**: Learning in an environment where information is encountered in sequences with *p* = 0 and all information thus is in single stimuli. **b**: Learning in an environment where information is encountered in sequences with *p* = 0.25. **c**: Learning in an environment where information is encountered in sequences with *p* = 0.5. **d**: Learning in an environment where information is encountered in sequences with *p* = 0.75. **e**: Learning in an environment where all information is encountered in sequences.

To evaluate the ability of a *Flexible sequence* representation to learn to recognize sequences with accuracy and efficiency, we simulate learning in an environment where the sequence (*A*, *B*) requires a different response from (*B*, *A*) (Fig 5E). In this environment, *A* and *B* also occur alone and intermixed with other stimuli, so that the sequences (*A*, *B*) and (*B*, *A*) cannot be identified by their first or last element alone. Here, a trace memory hardly learns to respond productively at all. While both *Depth-ℓ* and *Flexible sequence* representations support discrimination of (*A*, *B*) from (*B*, *A*), the *Flexible sequence* representation generates much faster learning (Fig 5E). Its flexibility allows for identification and symbolizing of relevant sub-sequences, so that they can be recognized independently of their temporal position. At the same time, it supports learning to ignore sub-sequences that are uniformative. For example, the *Flexible sequence*representation, differently from the original *Depth-ℓ* representation, perceives the similarity between the sequences (*A*, *B*, 0) and (0, *A*, *B*).

In four additional learning simulations we vary the probability *p* of information being in sequences and the probability 1 − *p* of information being in single stimuli (Fig 5A, 5B, 5C and 5D). When more information is in single stimuli, the *Flexible sequence* representation suffers higher learning costs than a trace memory, due to the fact that it considers a higher number of representations (see Fig 5). It is, however, much less costly than the *Depth-ℓ* representation, indicating that its ability to ignore irrelevant information trumps the fact that it generates more representations. In a pre-human evolutionary scenario without culture on a grand scale, we may assume that the order of stimuli is less important than the stimuli themselves, and information in sequences thus less frequent than information in single stimuli. In an example of such an environment, where one forth of the information is in sequences (Fig 5B), the *Flexible sequence* representation can have an evolutionary advantage over a trace memory, but only if learning time is relatively long.

## Methods

To explore a potential first step in the evolution of language we use both analytical modelling and computer simulations of learning. Here we describe the method of the computer simulations and briefly the relation between the simulations and the analytical model.

### Simulations

In the computer simulations, learning occurs by a simple and traceable error-correction function, theoretically equivalent to current models of learning [100–102]. A deep network is not necessary for our aims, as we are interested in the process of learning to discriminate, and not stimulus generalization. We simulate learning about a binary decision, such as deciding whether to eat or not eat a bug based on feedback about it being edible or not. In the simulations, an organism interacts with an environment and learns at each interaction. The interactions occur at discrete time-steps, and a simulation runs in a pre-assigned number of time-steps (or learning opportunities). At each time-step the agent is exposed to a sequence of stimuli, performs a behavior as a response to the sequence, and learns from the consequence of that behavior. Decision-making and learning occur according to equations that are well grounded in experimental psychology and machine learning [103–106]. The learning simulations and the underlying equations are specified in S1 File. After a number of time-steps the performance of the agent in the environment is measured. The analytical model which is presented below follows similar principles when analysing the learning costs of sequence representation in the sense that learning occurs in time-steps governed by mathematical assumptions about the rate of learning and that learning occurs in an environment where the temporal distribution of information is specified. In the simulations, the following is performed at each time-step:

1. A sequence is drawn from the possible sequences in the environment (see *The environments* below).
2. An internal representation of this sequence is created. This representation differs between the memory strategies (see *Representations* below).
3. The agent responds to the sequence using the response function described in *Representations* below, and as a consequence receives a reinforcement value that depends on the response and whether the sequence is rewarding or not (see *The environments* below).
4. This reinforcement value is used to update the associative strengths for this response [102] (see also Equation 2 in the S1 File).
5. Every 100 steps, the agent’s performance is measured. This is done by “freezing” the simulation time-steps and letting the agent respond to a fixed set of “test sequences”. The fraction of correct responses to these test sequences is measured and recorded. The exposure to the test sequences does not affect the associative strengths that are updated in point 4.

Then the next sequence is drawn, and so on.

### The environments

An environment consists of a number of informative stimuli and a number of noise stimuli. The set of possible sequences of these stimuli in the environment is constructed through a number of template sequences. Each position in a template sequence is either an *informative* stimulus or a *noise* (noninformative) stimulus. For example, each sequence of symbols in Fig 3A represent a template sequence in an environment with two informative stimuli ● and ◯ where ✲ indicates a noise stimulus. Thus, the template ✲✲✲● represents all sequences starting with three noninformative stimuli followed by one of the informative stimuli.

In each time-step of the simulation, one of the template sequences is picked uniformly at random, and each of its noise positions are replaced by one of the noise stimuli, chosen uniformly at random. Each template sequence is either rewarding or nonrewarding. These are constructed such that exactly half of the template sequences are rewarding and half nonrewarding.

### The agent

The agent’s behavior repertoire is limited to the two behaviors *go* and *no-go*. The agent receives the highest reinforcement value (5) when responding to a rewarding sequence, and the lowest (−4) when responding to a nonrewarding sequence, and no reinforcement (0) when not responding (regardless of stimulus sequence). The negative reinforcement value represents the cost of performing a behavior that does not render any utility. This cost is naturally lower than the utility gained by peforming the correct behavior.

### Representations

In this paper we evaluate different strategies for sequence representation. Below follows a formal description of the representations considered in the manuscript. Each representation strategy has a particular way of representing the incoming stimulus sequence. This representation is used in the decision function and in the equation that updates the associative strengths when learning.

The representation feeds information into the decision function and the memory updating equation. We here define these equations for the different representations. In our simulations the sequences have length four. Thus, consider a stimulus sequence *D*, *C*, *B*, *A*. Each representation strategy represents this sequence as a set *P* of *perception elements*. Each element *p* = (*K*, *x*) ∈ *P* consists of (I) a subsequence *K* of the stimulus sequence *D*, *C*, *B*, *A*, and (II) an intensity *x* of that subsequence. In the representation *Trace*, each subsequence is simply one of the stimulus elements (*A*, *B*, *C*, or *D*), with a geometrically decaying intensity. In *Depth-ℓ*, there is only one perception element where the subsequence is the entire percieved sequence. In *Flexible sequence of depth-ℓ*, all possible subsequences are present in *P*. We have the following perception elements after experiencing *D*, *C*, *B*, *A*.

- *Trace*: (*D*, *θ*^3^), (*C*, *θ*^2^), (*B*, *θ*), (*A*, 1)
- *Depth-1*: (*A*, 1)
- *Depth-2*: (*BA*, 1)
- *Depth-3*: (*CBA*, 1)
- *Depth-4*: (*DCBA*, 1)
- *Flexible sequence of depth-4*: $\begin{array}{c} (DCBA,1),\;(DCB,1),\;(CBA,1),\\ (DC,1),\;(CB,1),\;(BA,1),\\ \left(D,1),\;(C,1),\;(B,1),\;(A,1\right) \end{array}$

## General discussion

Language requires accurate sequence representation. Here, we have shown that such representations are unlikely to evolve because they incur high learning costs due to a combinatorial explosion associated with sequential information. In addition, a trace memory (found in most animals) [23] represents an efficient solution for taking past information into account, while avoiding the abovementioned combinatorial explosion. In situations where representing the exact order of arbitrary stimuli is not necessary, as may be mostly the case for non-human animals, a trace memory is more efficient than more accurate sequence representation. However, if information is structured sequentially so that the order of stimuli is meaningful, a trace memory proves to be insufficient and a more accurate sequence representation is necessary. The learning costs induced by the combinatorial explosion still need to be avoided, making strategies for excluding unnecessary information important. Learning to symbolize relevant sequences, so that they can be easily recognized and remembered is one such strategy, and learning to delete information of little interest from representations is another. A simple example of a representation that allows for such strategies is a flexible sequence representation that considers recently perceived sub-sequences, rather than considering the whole information stream as one unique sequence. This flexible sequence representation can also be considered cognitively plausible given that human working memory can process single elements as well as different combinations of elements [96].

If a sufficiently large proportion of information is structured sequentially and an organism invests heavily in learning, then this kind of flexible sequence representation may be favored by natural selection. These conditions are unlikely to be fulfilled among animals but may have occurred in human ancestors, considering that large primates learn throughout an extensive juvenile period and that, for example, manufacturing and use of tools may have increased the amount of sequentially structured information in early human evolution [25]. Tentatively, the evolution of accurate and flexible sequence representation may have set the stage for the emergence of language and other mental phenomena that underlie cumulative culture, for instance planning, thinking and sharing symbols [12, 23], in their turn favouring increased learning time. Such a gene-culture co-evolutionary scenario is compatible with life-history evolution of a uniquely long human childhood [107].

Previous models of co-evolution of language and cognition tend to give a larger role to biology. It has been suggested that specific learning biases evolved to adapt to characteristics of existing languages [9, 108]. Others have applied evolutionary game theory to explore how an expanding vocabulary generated by the capacity for combining sounds creates a selective pressure for compositional grammar [109–111]. These proposals have in common that they assume unusually stable linguistic environments, and postulate that specific genetic adaptations facilitating language acquisition would evolve in such environments. We propose a more general and plausible co-evolutionary trajectory relying on sequence representation as a first crucial step, where extended learning time is an additional adaptation that facilitates the acquisition of increasingly complex language, as well as other culture. Furthermore, while we agree with the idea of compositional grammar emerging as a solution for managing the combinatorial explosion generated by a large vocabulary, we propose that this emergence would result from cultural and not genetic evolution, relying upon the foundation of accurate and flexible sequence representation.

In the longstanding debate on whether the difference between humans and other animals is of a degree or a kind [112, 113], our results favour the hypothesis that humans evolved a new kind of sensitivity to sequential order, a small but significant step, that could give rise to the gradual emergence of mental skills and language.

## Supporting information

S1 File Supplementary material. The supplementary material contains some additional information on the analytical model and the computer simulations presented in this manuscript. It also includes a link for downloading the python script used for performing the simulations and a brief description of the script.
(PDF)
