# Supplementary material for: Sequence representation as an early step in the evolution of language
Source: PLoS Comput Biol. 2023 Dec 13;19(12):e1011702. doi: 10.1371/journal.pcbi.1011702 (PMC10752568; doi:10.1371/journal.pcbi.1011702)
Supplement: S1 File — The supplementary material contains some additional information on the analytical model and the computer simulations presented in this manuscript. It also includes a link for downloading the python script used for performing the simulations and a brief description of the script. (PDF) [file pcbi.1011702.s001.pdf]

Supplementary material to *Sequence representation as an early step in the evolution of language*

Anna Jon-And[1,2], Markus Jonsson[1], Johan Lind[1], Stefano Ghirlanda[1,3,4], & Magnus Enquist[1,5]

October 20, 2023

1. Centre for Cultural Evolution, Stockholm University, Sweden
2. Department of Romance Studies and Classics, Stockholm University, Sweden
3. Department of Psychology, Brooklyn College of CUNY, Brooklyn, NY, USA
4. Department of Psychology, CUNY Graduate Center, New York, NY, USA
5. Department of Zoology, Stockholm University, Sweden

This manuscript is based on an analytical model and computer simulations. The analytical model is presented in the manuscript and here we present some additional information. The computer simulations were performed using a python script and here we describe the script briefly and present complementary results. This supplementary information also includes a link for downloading the script.

Supplementary text

Analytical model

Figure S1 shows that the result in Figure 1 in the manuscript (that the learning of sequential information is expensive in terms of learning opportunities), holds also when varying the parameters τ (the average number of learning opportunities required to learn a given sequence) and r (controlling the rate by which the proportion of informative sequences in the world grows with sequence length).

As we see, considering past stimuli is favored only if the amount of information in the world increases quickly with decision depth (small r), or if there is a lot of time for learning (large T).

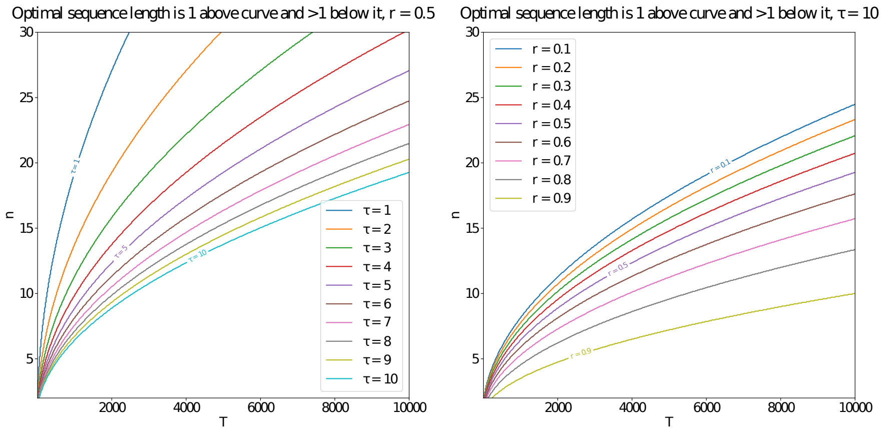

Figure S1. Effect of varying τ and r on the optimal decision depth. For example, in (a) we see that even in a world with as few stimuli as 30, and when each sequence is learned immediately ($\tau = 1$), more than 2,000 learning opportunities are required for the optimal depth to be > 1.

Simulations

Decision-making and learning in the simulations occur according to the following principles. The associative strengths $v(p \to B)$ are indexed by pairs (perception element p, behavior B). After exposure to a sequence, decision making is based on the perceptual elements P using a softmax rule (Sutton & Barto 2015) wherein the probabilities for the behaviors G and N as the response to the sequence are computed as follows:

$$\text{Prob}(P \to \text{G}) = \frac{s(\text{G})}{s(\text{G}) + s(\text{N})}, \quad \text{Prob}(P \to \text{N}) = \frac{s(\text{N})}{s(\text{G}) + s(\text{N})} \tag{1}$$

where $s(\text{B}) = \exp\left(\beta \sum_{p' \in P} x(p')\, v(p' \to \text{B})\right)$ and β is the exploration factor where increasing β decreases the amount of exploration. The response B (either G or N) is then drawn using these probabilities. Then, for each $p \in P$, the associative values $v(p \to B)$ are updated according to

$$\Delta v(p \to \text{B}) = \alpha \left[u - \sum_{p' \in P} x(p')\, v(p' \to \text{B}) \right] x(p) \tag{2}$$

where u is the reinforcement value and α the learning rate.

The program

Description of the Python script

The Python script `make_figures.py` The function `fig_panel_in_analytical_model` produces Figures 1b and 1c in the manuscript. The function `fig_tau_r_supplementary` produces the charts in Figure S1 in this supplementary material. Both of these are well-commented and should be clear based on the definitions in the section “Benefits and costs of sequential information” in the manuscript.

The function `fig_nature_parameter_study` produces the panels in Figures 2b, 2c, and 2d.

The function `fig_culture` produces Figure 3.

Download script

The Python script `make_figures.py` is available at `https://github.com/markusrobertjonsson/firststep`.
